# Efficacy of Dietary Supplementation with Zinc-Chromium Mixture, Organic Selenium, or Their Combinations on Growth Performance, Carcass Traits, and Blood Profiles of Broilers under Heat Stress Conditions

**DOI:** 10.3390/ani13152539

**Published:** 2023-08-07

**Authors:** Abdelhameed S. A. Mohamed, Maha A. Abd El Latif, Eman A. M. Hussein, Enas M. A. Toson, Mohamed Saleh, Dariusz Kokoszynski, Shaaban S. Elnesr, Mohamed Mohany, Salim S. Al-Rejaie, Hamada Elwan

**Affiliations:** 1 Animal and Poultry Production Department, Faculty of Agriculture, Minia University, EL-Minya 61519, Egypt; abdalhmed.salah@mu.edu.eg (A.S.A.M.); maha.omr@mu.edu.eg (M.A.A.E.L.); enas.toson@mu.edu.eg (E.M.A.T.); 2 Department of Poultry and Fish Production, Faculty of Agriculture, Menoufia University, Shibin El-Kom 32514, Egypt; eman.hussien@agri.menofia.edu.eg; 3 Department of Poultry Production, Sohag University, Sohag 82524, Egypt; bydg2016@gmail.com; 4 Department of Animal Breeding and Nutrition, Bydgoszcz University of Science and Technology, 85084 Bydgoszcz, Poland; kokoszynski@gmail.com; 5 Department of Poultry Production, Faculty of Agriculture, Fayoum University, Fayoum 63514, Egypt; ssn00@fayoum.edu.eg; 6 Department of Pharmacology and Toxicology, College of Pharmacy, King Saud University, P.O. Box 55760, Riyadh 11451, Saudi Arabia; mmohany@ksu.edu.sa (M.M.); rejaie@ksu.edu.sa (S.S.A.-R.)

**Keywords:** broiler, chromium, zinc, selenium, blood

## Abstract

**Simple Summary:**

The need for methods to lessen the impacts of heat stress has increased due to climate change. The addition of trace elements to the food of chicks is an approach used to enhance production yield while boosting grill productivity. In livestock production, selenium is a crucial trace mineral for improved performance, physiological state, and health advantages. Numerous studies have examined the advantages and uses of both organic and inorganic selenium in poultry feeding. On the other hand, there is a dearth of knowledge on the use of organic selenium in heat-stress situations. In this work, we assessed the impact of dietary organic selenium on broiler growth performance, carcass characteristics, and blood profiles under heat stress, with or without a zinc-chromium mixture. According to the findings, adding organic selenized yeast improves the haematological, biochemical, and antioxidant status of broilers as well as their productivity. The weights and carcass qualities of the birds given Zn-Cr plus low or high Se levels were improved. In light of this, we concluded that the combined effect of feed additives (Zn-Cr and organic-Se) could reduce the effects of heat stress on broilers by increasing metabolic processes and enhancing growth performance.

**Abstract:**

To determine the effects of organic selenium (0.0–0.6 mg and 0.9 mg Se/Kg diet) and Zn-Cr mixture (100 mg Zn/Kg diet plus 1.5 mg Cr/Kg diet) on broiler chicken performance, carcass traits, blood hematology, and biochemistry under heat stress conditions, this study was conducted. Under temperatures between 30.21 to 31.82 °C, 240 broiler chickens (Ross-308), which were 7-day-old, were randomly assigned to one of six treatments: T1 (control), T2 (100 mg Zn per kg of diet and 1.5 mg Cr per kg of diet), T3 (0.6 mg Se per kg of diet), T4 (0.9 mg Se per kg of diet), T5 (100 mg Zn, 1.5 mg Cr and (LSe), and T6 (100 mg Zn, 1.5 mg Cr and (HSe)). At 35 days old, the chicks fed a diet containing Zn-Cr with low or high organic selenium (organic-Se) outweighed the control group in terms of live body weight, weight gain, and feed conversion ratio (*p* < 0.05). In comparison to the control treatment, birds fed diets supplemented with Zn-Cr or organic-Se (LSe, HSe) significantly increased their serum levels of total protein and total antioxidant capacity. However, these additives resulted in a decrease (*p* < 0.01) in their serum levels of triglycerides, total cholesterol, low-density lipoprotein (LDL) cholesterol, creatinine, and uric acid. Together, it was found that trace elements (Zn-Cr and organic-Se) may greatly lessen the impacts of heat stress on broilers by promoting growth performance and boosting metabolic processes.

## 1. Introduction

Heat stress affects the performance of birds [1]. This HS (≥30 °C) alters the physiological biochemistry of birds, resulting in a high drop in feed intake and feed efficiency, which harms these birds’ performance and productivity [2,3]. Therefore, to minimize the adverse effects of HT on contemporary strains of broilers, it is crucial to regularly improve the nutritional techniques and implement optimal feeding methods in sync with the rapid growth of these birds, as demonstrated by the preceding explanation of the significance and influence of HT on them.

Feed additives are used in broiler diets to improve digestive efficiency. Nutritional advances in feed additives include plant extracts, synthetic amino acids, enzymes, trace minerals, vitamins, prebiotics, probiotics, etc. [4,5,6,7]. Minerals play a vital role as nutraceuticals in poultry production because they are required for birds’ optimum physiological and metabolic responses. In broiler chickens, minerals are an essential part of the activation of enzymes and hormones, acid-base balance, and osmotic homeostasis [1].

Currently, Cr is thought to have a substantial impact on physiological and nutritional processes in addition to being regarded as an essential trace element for broilers [8]. While Cr plays the main role in protein, lipid, carbohydrate, and nucleic acid metabolism because it influences insulin, adding Cr to the diet of chickens could enhance glucose transport and reduce temperature stress by decreasing stress protein levels [9].

Zinc is one of the most crucial trace minerals for nutrition and a crucial part of many biological processes [10]. Zinc has a vital role in metalloenzymes and has both structural and catalytic functions. It is essential for healthy development and physiological processes in the body and plays a critical antioxidant role in the body’s antioxidant defense system [11]. Zinc promotes healthy bones, efficient growth, and hunger control in chickens [12]. Selenium (Se) is a crucial trace element in livestock production for better performance, higher-quality meat, and other health advantages. In either inorganic or organic form, selenium has been added to broiler meals to satisfy the needs of the birds [13]. Selenium is a crucial trace element for poultry rearing at high temperatures by participating in several biochemical pathways and physiological functions as a vital part of a range of important selenoproteins that are related to body metabolism, cellular homeostasis, and antioxidant defense [14,15]. Naturally, Se can be present in two main chemical forms: (1) organic form found in tissues of animals and natural plants that are typically selenocysteine (SeCys), selenomethionine (SeMet), and Se-enriched yeast (SY); and (2) inorganic form including selenate, selenite, and selenide [16]. There has recently been interest in using organic forms of Se, which are less toxic and have greater efficacy than inorganic forms, on poultry under HT. SY is one of the organic forms and is mostly made of SeMet and other Se compounds at low concentrations [15]. While the toxic levels of Se in broiler diets are higher than 15 mg/kg [17], also adding Zn-Cr to broiler diets up to 3, 160 mg/kg, respectively, can improve productive performance and the physiological state of these chicks [18,19].

It has been shown that adding trace elements to broiler diets can have a variety of advantageous effects on broiler production [20,21,22,23]. A combination of Zn, Se, and Cr supplementation can improve the tissues’ use of glucose and boost protein synthesis, both of which improve broiler growth performance [23]. Previous studies have shown that supplementing broiler diets with trace minerals (Zn, Cr and Se) boosts performance and immunity under heat stress [24]. We sought to identify the highest concentrations of Zn-Cr combination and organic-Se (LSe, HSe) for broiler diets under heat stress based on our prior results [25,26]. Our current research aims to continuously examine the effects of these supplements on broiler blood profiles, carcass traits, and growth performance under heat-stress conditions.

## 2. Materials and Methods

### 2.1. Chicks and Management

The Animal and Poultry Production Department farm at Minia University, Faculty of Agriculture, experimented with 7-day-old mixed-sex chicks (Ross-308, *n* = 240). In an open-sided house, chicks were reared in floor pens during the whole growing period (7–35 days of age), and all pens were bedded with wheat straw. Broiler chicks were brooded (41.92 ± 0.47 g) together from day 1 to day 7 of age. On day 7, chicks were weighed and randomly distributed (with an initial live body weight of 154.09 ± 4.7 g) into six experimental groups of 40 chicks with four replicates of 10 chicks each based on 10 chicks/m^2^. The light and dark cycles were 23 h (h) and 1 h, respectively, per day throughout the whole growing period. To ensure a consistent blend, the feed ingredients were carefully incorporated by hand and thoroughly mixed. Additionally, the supplements were meticulously combined with the basal diet, guaranteeing uniformity. The supplements were added above the basal diet and mixed for a specific duration, ensuring proper distribution throughout. Then, the diets were crumbled and fed as starter diet from d 7 to d 21, and a finisher diet from d 22 to d 35 according to Aviagen, [27], with free access to feed and water. The chemical composition (Table 1) was determined according to the Association of Official Analytical Chemists methods [28]. Chicks in T1 (control), were fed on basal diets with no additive; however, chicks in T2, T3, T4, T5, and T6 were fed on basal diets with Zn and Cr (Zn-Cr), and organic-Se at different levels (Table 2).

The ambient temperatures ranged from 30.21 to 31.82 °C during the trial period, with relative humidity (RH) values ranging from 67.64 to 70.57%. The temperature was about 5–10 °C higher than recommended by the Ross Management Guides [29]. Every 2 h, the ambient temperatures and RH were noted daily (Table 3).

### 2.2. Mortality Rate and Growth Parameters

Every week, feed consumption (FC) and live body weight (live BW) were recorded. For dead birds, FC was modified. Feed conversion ratio (FCR) and body weight gain (BWG) were calculated. FCR was determined using the kilogram (kg) of FC per 1 kg of BWG and then adjusted for the number of broiler chicks in each replicate.

### 2.3. Carcass Traits

The carcass features of two broilers from each replicate (8/treatment) were estimated by weighing them after a 12 h fast on day 35 of age. The feathers were removed (via a ventral cut) and plucked apart. The gizzard, liver, heart, abdominal fat, and spleen are all separately weighed and expressed as a relative weight (g/100 g of live BW). The carcass weight (without the head, shanks, and visceral organs) is also shown.

### 2.4. Haematological and Biochemical Measurements

On day 35, two blood samples per replicate were withdrawn (8 samples/treatment) by the wing vein in two blood-collected tubes (with or without heparin). A heparinized tube (as an anticoagulant) was used for measurement of red blood cells (RBCs), white blood cells (WBCs), hemoglobin (Hb), and packed cell volume (PCV). Red blood cells and WBCs were determined using a hemocytometer. Hemoglobin concentration (g/dl) was estimated by the cyanmethemoglobin method and PCV (%) by micro-hematocrit centrifuge. The non-heparinized tubes were kept at room temperature for 20 min to clot. After that, samples were centrifuged for 15 min at 3000 rpm to separate the serum. Total protein (TP), albumin (Alb), glucose (Glu), alanine and aspartate aminotransferases (ALT and AST), creatinine (Creat), uric acid (UA), triglycerides (Trig), total cholesterol (TC), high-density lipoprotein cholesterol (HDL-cholesterol), low-density lipoprotein cholesterol (LDL-cholesterol), total antioxidant capacity (TAC), and malondialdehyde (MDA) levels were measured in the serum samples that were kept at −20 °C. Using commercial kits from Diagnostic, Egypt, these parameters were ascertained. Using kits obtained from Diagnostic Products Corporation (Los Angeles, CA, USA), serum triiodothyronine and thyroxine concentrations were estimated.

### 2.5. Statistical Analysis

All data are presented as mean ± SE. The two-way factorial design (2 × 3) was used to establish the statistical analysis processing of the data. This design was applied to assess the interaction between the main effects elements (dietary Zn-Cr and organic-Se) by applying the SAS program (version 9.4, 2013). Duncan’s test was used to determine the significance of the mean differences. The probability was considered significant at *p* < 0.05.

## 3. Results

### 3.1. Growth Performance

Growth performance indicators, including live BW, BWG, and FCR (except FC) of broiler chickens given supplemented diets were significantly improved (*p* < 0.01) during broiler chick growing periods (Table 4). During the starter and the finisher periods the individual effect of adding Zn-Cr or organic-Se (LSe, HSe) to broiler diets markedly enhanced (*p* < 0.001) live BW, BWG, and FC; however, no significant changes (*p* > 0.05) were observed in live BW and FCRs by adding organic-Se through the starter period, compared with the control group under HT. Regarding the interaction between Zn-Cr and organic-Se, the largest BW and BWG were found in T5 (Zn-Cr with LSe) and T6 (Zn + Cr with HSe), followed by T2 (Zn-Cr). With the same trend, these treatments (T5, T6 followed by T2) recorded the best FCRs, compared to the other treatments and the control.

### 3.2. Carcass Traits

The results presented in Table 5 show the effects of supplementation of organic-Se and Zn-Cr individually or combined on carcass yield and organ percentages at d 35 of age. Broiler chickens fed the diet with Zn-Cr alone presented greater (*p* < 0.01) carcass yield, gizzard, liver, heart, and abdominal fat percentages, but the spleen % of these supplemented chicks did not change (*p* > 0.05), compared with un-supplemented chicks. In contrast, adding organic-Se (LSe, HSe) alone had no significant effect (*p* > 0.05) on carcass characteristics, compared with the control. Data revealed that the interaction effect between Zn-Cr and different levels of organic-Se (LSe, HSe) improved (*p* < 0.0001) carcass yield, liver, and abdominal fat; however, no significant changes were documented (*p* > 0.05) in gizzard, heart, and spleen percentages. The highest values (*p* < 0.01) of carcass yield (%) and liver (%) were noticed in T5 (Zn-Cr-LSe) and T6 (Zn-LSe) followed by T2 (Zn-Cr). Moreover, abdominal fat (%) statistically decreased in the same treatments compared to other supplemented or un-supplemented treatments under heat stress.

### 3.3. Haematological Characteristics

The influence of Zn-Cr, two levels of organic-Se supplementation, and their interaction on red blood cells (RBCs), white blood cells (WBCs), hemoglobin (Hb), and packed cell volume (PCV) in broiler chickens is reported in Table 6. Adding Zn-Cr alone led to an increase (*p* < 0.001) in RBC, Hb, and PCV; however, it did not affect WBC values. Nevertheless, there are no significant results by supplementation organic-Se on hematological indices. The interaction effects of Zn-Cr × organic-Se content were significantly increased (*p* < 0.01) for RBC, Hb, and PCV but were not changed (*p* > 0.05) for WBC, compared with the CON group. Chicks In T2, T5, and T6 (Zn-Cr, Zn-Cr-LSe, Zn-Cr-HSe, respectively) recorded the highest levels of RBC, Hb, and PCV compared to the other two treatments (LSe, HSe) and the control.

### 3.4. Serum Blood Biochemistry

Data regarding the serum blood biochemistry of broiler chickens fed the experimental diets are presented in Table 7. Birds that were fed diets with Zn-Cr alone had markedly increased (*p* < 0.001) values of serum TP, Glu, and HDL-cholesterol and reduced (*p* < 0.001) Alb, Trig, TC, LDL-cholesterol creatinine, and uric acid; however, the values of ALT and AST in serum did not differ (*p* > 0.05) compared with chicks fed the control diet. The individual effects of adding organic-Se (LSe, HSe) statistically decreased (*p* < 0.01) serum LDL-cholesterol and UA but no remarkable changes (*p* > 0.05) were noticed in serum TP, Alb, Glu, TC, HDL-cholesterol, ALT, AST, and creatinine. While serum blood chicks fed diets with HSe had lower Trig (*p* < 0.05), compared to those fed diets with LSe or fed diets without the addition of Se (CON). Data revealed that the interaction effect between Zn-Cr and organic-Se (LSe, HSe) on serum parameters was significant (*p* < 0.01), but no differences (*p* > 0.05) were observed in ALT and AST, compared with the control. Supplemented treatments (T2, T5, and T6) recorded the highest TP, Alb, HDL-cholesterol, and the lowest Glu, Trig, TC, LDL-cholesterol, Create, and UA, compared to other treatments.

### 3.5. Thyroid Hormones

Both Zn-Cr and organic-Se (singly or in combination) had a significant effect on T3 but did not affect thyroxine at d 35 (Table 8). Supplemented diets with Zn-Cr or LSe, enhanced (*p* < 0.05) serum triiodothyronine; however, there was no significant effect of HSe on serum triiodothyronine, compared with the control group. Furthermore, the highest recorded (*p* < 0.001) activity of triiodothyronine was noted in treatment T5 (Zn-Cr with Lse), compared with other treatments or the control group.

### 3.6. Serum Antioxidant Activity

Serum TAC and MDA were markedly affected (*p* < 0.01) by Zn-Cr and/or organic-Se (Table 9). The influence of Zn-Cr alone clearly (*p* < 0.01) improved serum TAC; however, there were non-significant decreases in serum MDA. Similarly, the addition of organic-Se (LSe, HSe) significantly improved TAC and reduced (*p* < 0.01) MDA compared with the control. The influence of Zn-Cr and/or two levels of organic-Se supplementation noticeably improved serum TAC and markedly decreased MDA in broiler chickens.

## 4. Discussion

The addition of Zn-Cr, organic-Se, and their interactions improved the BW and BWG of broiler chicks maintained at high temperatures. Throughout the entire period (8–35 days), chicks in T5 and T6 (Zn-Cr with Se) had a better performance in comparison with other groups. This result is in line with that of Rao et al. [21] who found that supplementing Se, Cr, and Zn (0.30, 2, and 40 mg/kg, respectively) in organic form boosted body mass gain considerably when compared to individuals on a control diet. Similarly, Marković et al. [30] found that 0.9 mg Se/kg supplementation boosted body weight and weight gain more than the control group. Previous research has confirmed these findings of Ramiah et al. [31]. Contrarily, it was shown that supplementing broilers with Se either alone or in combination with other trace minerals did not affect growth performance [32]. The improvement in BWG may be attributed to the usage of chelated minerals which can boost intestinal trace element absorption by reducing interference from substances that generate insoluble complexes with the ionic trace elements, thus improving their bioavailability and BW development [33,34]. Or, maybe it is because dietary supplements improve feed utilization and nutrient absorption compared to broilers fed a control diet while under heat stress (HT), which had poor growth performance and used more energy to adapt to the stressor than they did for growth [35].

Supplementing LSe (T5) and 100 mg Zn + 1.5 mg Cr reduced FC and improved FCRs considerably. Our results are in line with those of Abdallah et al. [36], who discovered no appreciable differences in feed intake in broiler diets supplemented with the chelated forms of Zn, Cu, Mn, and Fe at 50 or 100% of total demands. In a similar study, Baloch et al. [37] found that organic trace element supplementation did not affect broiler feed consumption. Contrary to the present findings, Yaqoob et al. [38] reported that dietary interventions did not impact total FC in broiler breeders when inorganic trace elements were replaced with complexed glycinates. No appreciable differences in feed efficiency were observed when broilers were fed diets containing 0.20 ppm Se from sodium selenite or selenium yeast [39]. Jain et al. [33] also discovered that Zn-Cr and Se improved FCR. Zinc supplementation improves nutrient digestibility and usage efficiency, which leads to better FCR and has a positive impact on feed conversion [40]. Additionally, Zinc shields pancreatic tissue from oxidative damage; as a result, it may aid in proper pancreatic function, including the secretion of digestive enzymes, improving nutrient performance and digestibility. The role of chromium in the metabolism of glucose, lipids, proteins, and nucleic acids is associated with its growth-promoting effects [41]. Additionally, it has been demonstrated that Cr stimulates the biological function of insulin-sensitive cell receptors by increasing their binding activity. Se-supplemented meals may be linked to the immunomodulatory effects of Se as well as greater serum concentrations of the thyroid hormone’s active form in chickens.

Supplementing broiler diets with Zn-Cr and Se (except for the effect of Se alone) increased carcass production and reduced abdominal fat. This result is in line with that of Jain et al. [33] who discovered that the combined effects of Zn, Se, and Cr resulted in heavier weights of carcass parts than the control group. They also discovered that dietary Cr caused a significantly higher carcass dressing percentage in broiler chickens that were given Cr supplements compared to the control group [42]. Furthermore, Huang et al. [43] discovered that supplementing broiler chicks with Cr under HS improved the dressing % in comparison to un-supplemented chicks. This may be because chelated trace minerals with growth-promoting properties had a synergistic effect on broilers’ dressed, eviscerated, and drawn weights. Comparing broiler carcass output to controls, Baltić et al. [44] and Khatun et al. [45] observed that organic trace minerals or Se supplementation had no effect.

Blood hematological parameters (except WBC) of chicks aged 35 days differed significantly when adding Zn-Cr either alone or in a mixture with organic-Se. In comparison to the other groups and the control group, birds in T2, T5, and T6 had the highest values of RBC, Hb, and PCV, while all treatment groups had RBC counts in the usual range of 2.5–3.5 × 10^6^/mm^3^. The increased immunity owing to the individual or combination of antioxidants shielding the chicken from pro-inflammatory and/or oxidative stress indicators in this study might be ascribed to the increased RBC of birds given supplemented meals [46]. The substantial variations in Se, Zn, and Cr on Hb % may be owing to the good influence of Se on hematological parameters, such as enhanced blood parameters or higher PCV, which will boost Hb %. The importance of Zn in erythropoiesis might explain the rise in hemoglobin in zinc-supplemented broiler chickens. Zn is also a catalytic component of the enzyme alfa-aminolevulinic acid dehydrogenase, which is involved in hem production [47]. The antioxidant properties of Zn-Cr and Se, as well as their capacity to boost the synthesis, stability, and activity of enzymes in the body, might explain the rise in hemoglobin in treatment groups supplemented with them singly or in combination. The rise in PCV % in supplemented groups with Zn and Cr, either separately or together, is likely due to their antioxidant characteristics and increased immunity.

The current findings showed that combining Zn-Cr and organic-Se enhanced blood biochemistry and metabolic responses (high total protein, low glucose, low triglycerides, low LDL cholesterol, high HDL cholesterol, low creatinine, and low uric acid). Our findings are consistent with those of other researchers. Saleh et al. [48] found that adding Zn methionine to heat-stressed broiler diets at doses of 25, 50, and 100 mg/kg resulted in a significant reduction in plasma triacylglycerol and TC concentrations when compared to the control group, while AST and Glu parameters remained unchanged. According to Arif et al. [49], Cr-propionate supplementation lowered serum Glu in broiler chicks as compared to the control group but did not affect liver enzymes (AST and ALT). Se can decrease the formation of thiobarbituric acid-reactive substances and increase the activity of glutathione S-transferase and total sulphysulphydryl groups, an essential part of the enzyme glutathione peroxidase, of which the enzyme activity prevents lipid and protein oxidation [50]. Additionally, according to Saracila et al. [51], dietary supplementation with Zn-Cr had a smaller effect on Glu levels than Cr plus vitamin C. Hassan et al. [52] found that rabbits fed a diet containing Zn and Se-enriched and Se-enriched spirulina or a combination of them had high TP and HDL-cholesterol levels.

In the current experiment, the single addition of Zn-Cr (100 and 1.5 mg/kg diet, respectively) or LSe (0.6 mg/kg diet) markedly enhanced (*p* < 0.05) serum T3; however, supplementation with organic-Se (LSe, HSe) in combination with Zn-Cr had no significant effect on serum thyroxin compared to control. This might be because these substances act as antioxidants and lower free radical levels. As a result, they strengthen antioxidant defences, guard against oxidation and damage to cellular membranes, and increase the effectiveness of antioxidant action [33,43,53]. Additionally, the effects of Se supplementation on thyroid activity and the transformation of thyroxin into triiodothyronine were the best ways to increase metabolism and metabolic rate within the body, which could explain the rise in blood triiodothyronine and rise in glucose levels [54].

Supplementation with Zn-Cr and/or organic-Se led to a noteworthy enhancement in serum antioxidant activity by significantly increasing TAC and decreasing MDA concentrations compared to the un-supplemented group. Our findings are in agreement with several previous reports [1,52]. Additionally, Rao et al., [21] reported that supplementation of 0.3 mg of Se, 2 mg of Cr, and 40 mg of Zn/kg in the diet (organic form) individually or in combination significantly improved the antioxidant responses (superoxide dismutase, MDA) of broilers in the open-sided house during the summer season. By increasing the TAC and reducing the MDA of supplemented broilers, the positive benefits of dietary supplementation with Zn-Cr and organic-Se that were seen in the current study under heat treatment may be partially useful in minimizing the negative effects of heat stress. Zn functions as a potent indirect antioxidant component that is highly effective in preventing the production of free radicals and delaying oxidative processes [55]. This may result in a decrease in the metabolic need for resources for reducing stress and a shift in these nutrients towards building muscle [21], which is reflected in chick performance.

## 5. Conclusions

Overall, the results of this study demonstrated that, either individually or in combination, adding 100 mg Zn + 1.5 mg Cr + 0.6 or 0.9 mg Se from yeast to broiler chicks under heat stress could enhance growth performance, carcass characteristics, haematological characteristics, serum biochemical indicators, and antioxidant status. The low and high dosages of the organic selenium with Zn-Cr mixture did not significantly differ in broiler productivity or health. Therefore, it is advisable to use organic selenium at the lowest dosage possible.

## Figures and Tables

**Table 1:** Ingredient composition and nutrient analysis of the control diets.

Ingredient (g/kg)	Starter (7–21 d)	Finisher (22–35 d)
Yellow corn	566.5	610.0
Soybean meal, 44% crude protein	264.0	219.0
Corn gluten meal	100.0	100.0
Sunflower oil	28.5	32.0
Dicalcium phosphate	21.0	20.0
Sodium chloride	3.0	3.0
Limestone	13.0	12.5
Dl-methionine ^b^	1.0	0.5
Vitamin-mineral premix ^a^	3.0	3.0
Analytical composition		
Dry matter (DM)	918.9	917.8
Crude protein (CP)	227.3	208.4
Crude fiber (CF)	29.7	33.1
Ether extract (EE)	62.4	78.1
Calculated composition		
Metabolizable energy (MJ/kg)	13.01	13.33
Calcium	10.04	9.9
Available phosphorus	5.2	4.5
Methionine + Cystine	9.0	8.0
Zinc (Zn, mg/kg)	51.10	50.17
Selenium (Se, mg/kg)	0.22	0.21
Chromium (Cr, mg/kg)	0.04	0.04

^a^ Provided per kilogram of diet: vitamin A, 12,500 IU; vitamin E, 30 IU; vitamin D3, 4000 IU; vitamin B12, 3 mg; vitamin K, 2.3 mg; niacin, 65 mg; riboflavin, 8 mg; thiamine, 2.2 mg; pyridoxine, 4 mg; pantothenic acid, 24.3 mg; biotin, 0.25 mg; folic acid, 1.2 mg; choline, 600 mg; copper from copper sulfate, 7.5 mg; manganese from manganese oxide, 125.1 mg; iron from ferrous sulfate, 60 mg; zinc from zinc oxide, 110 mg; selenium from sodium selenite, 0.35 and iodine from ethylene diamine dihydride, 1.8 mg. ^b^ DL-Met, Met AMINO (DL-2-amino-4-(methyl-thio)-butane acid, DL-Met, α- amino-Y-methyl-oily acid) by Feed Grade 99%.

**Table 2:** Supplementation with some trace minerals (Zn-Cr and organic-Se) for broiler diets.

Treatments	Feed Additives, mg/kg Diet		
	Zn ^a^ (mg/kg Diet)	Cr ^b^ (mg/kg Diet)	Se ^c^ (mg/kg Diet)
T1 (control)	-	-	-
T2 (Zn-Cr)	100	1.5	-
T3 (LSe) *	-	-	0.6
T4 (HSe) **	-	-	0.9
T5 (Zn-Cr-LSe)	100	1.5	0.6
T6 (Zn-Cr-HSe)	100	1.5	0.9

^a^ = ZnSO_4_, ^b^ = chromium picolinate, CrPic; 12,42% and ^c^ = Selenium was derived from yeast (*S. cerevisiae*) in product ALKOSEL^®^ R397, Lallemand Inc., United Kingdom. * LSe = low selenium concentration, ** HSe = high selenium concentration.

**Table 3:** The temperature and relative humidity during the experimental period (8–35 days of age).

Parameter	Period (Days)			
	8–14	15–21	22–28	28–35
Ambient temperature, °C	31.82	31.69	30.45	30.21
Ambient temperature (12:00–16:00), °C	33.41	33.72	32.16	31.53
Relative Humidity (RH), %	70.57	69.81	67.64	67.91

**Table 4:** The impact of dietary minerals (Zn-Cr and organic-Se) supplementation on broilers growth performance under heat stress conditions.

Treatments	Live Body Weight, (g), Live BW			Body Weight Gain, (g), BWG			Feed Consumption, (g), FC			Feed Conversion Ratio, FCR		
	7	21	35	8–21	22–35	8–35	8–21	22–35	8–35	8–21	22–35	8–35
	(A) Effect of Zn-Cr mixture											
Without Zn-Cr	152.70	827.09 ^b^	1824.43 ^b^	674.39 ^b^	997.34 ^b^	1671.73 ^b^	1089.04	2074.80	3163.84	1.62 ^b^	2.09 ^b^	1.90 ^b^
With Zn-Cr	155.48	913.30 ^a^	1967.67 ^a^	757.82 ^a^	1054.36 ^a^	1812.19 ^a^	1097.80	2022.76	3120.56	1.45 ^a^	1.92 ^a^	1.72 ^a^
SEM	2.73	12.72	12.01	10.63	8.90	10.23	10.87	19.41	16.68	0.02	0.02	0.01
*p*-value	0.4814	0.0001	<0.0001	<0.0001	0.0003	<0.0001	0.5756	0.0742	0.0831	<0.0001	<0.0001	<0.0001
	(B) Effect of organic Se levels											
Without Se	151.74	852.82	1822.80 ^b^	701.08	969.98 ^b^	1671.06 ^b^	1094.55	2044.26	3138.81	1.57	2.12 ^a^	1.89 ^a^
With LSe ^≠^	153.46	873.01	1921.85 ^a^	719.55	1048.85 ^a^	1768.40 ^a^	1079.75	2048.16	3127.91	1.51	1.96 ^b^	1.77 ^b^
With HSe ^≠≠^	157.08	884.77	1943.49 ^a^	727.69	1058.72 ^a^	1786.41 ^a^	1105.96	2053.92	3159.89	1.53	1.94 ^b^	1.77 ^b^
SEM	3.34	15.58	14.71	13.01	10.90	12.53	13.31	23.78	20.42	0.02	0.02	0.02
*p*-value	0.5268	0.3619	<0.0001	0.3550	<0.0001	<0.0001	0.3962	0.9591	0.5421	0.1926	<0.0001	0.0001
	(A × B) Effect of interaction between Zn-Cr mixture and organic Se levels											
T1	149.91	807.77 ^c^	1714.22 ^d^	657.86 ^b^	906.45 ^c^	1564.31 ^e^	1080.06	2056.16	3136.22	1.65 ^a^	2.27 ^a^	2.01 ^a^
T2	153.57	897.87 ^ab^	1931.38 ^ab^	744.30 ^a^	1033.51 ^ab^	1777.81 ^bc^	1109.04	2032.36	3141.40	1.49 ^b^	1.97 ^bc^	1.77 ^c^
T3	152.63	832.50 ^bc^	1853.64 ^c^	679.87 ^b^	1021.14 ^b^	1701.01 ^d^	1086.53	2062.48	3149.01	1.60 ^a^	2.02 ^b^	1.85 ^b^
T4	155.57	841.01 ^bc^	1905.44 ^bc^	685.44 ^b^	1064.43 ^ab^	1749.87 ^cd^	1100.52	2105.78	3206.30	1.61 ^a^	1.98 ^bc^	1.83 ^bc^
T5	154.28	913.51 ^a^	1990.07 ^a^	759.23 ^a^	1076.56 ^a^	1835.79 ^a^	1072.96	2033.85	3106.81	1.42 ^b^	1.89 ^c^	1.69 ^d^
T6	158.59	928.53 ^a^	1981.55 ^a^	769.94 ^a^	1053.02 ^ab^	1822.96 ^ab^	1111.40	2002.08	3113.48	1.45 ^b^	1.90 ^c^	1.71 ^cd^
SEM	4.73	22.03	20.81	18.41	15.42	17.72	18.82	33.63	28.88	0.03	0.03	0.02
*p*-value	0.8569	0.0046	<0.0001	0.0012	<0.0001	<0.0001	0.6220	0.3992	0.2382	0.0002	<0.0001	<0.0001

^a,b,c,d,e^ = means with different letters in the same column are significantly different at (*p* < 0.05). SEM, standard error of the mean. ^≠^ LSe = low selenium concentration, ^≠≠^ HSe = high selenium concentration. T1 = (basal diet, no additive), T2 = T1 + 100 mg Zn + 1.5 mg Cr/Kg diet (Zn-Cr), T3 = T1 + 0.6 mg Se/Kg, T4 = T1 + 0.9 mg Se/Kg, T5 = T1 + (100 mg Zn + 1.5 mg Cr + 0.6 mg Se) and T6 = T1 + (100 mg Zn + 1.5 mgCr + 0.9 mg Se).

**Table 5:** The effect of dietary minerals (Zn-Cr and organic-Se) supplementation on carcass traits of broilers under heat stress conditions. (*n* = 8).

Treatments	Body Weight, g	(g/100 g of Live Body Weight)					
		Carcass Yield	Gizzard	Liver	Heart	Abdominal Fat	Spleen
	(A) Effect of Zn-Cr mixture						
Without Zn-Cr	1815.42 ^b^	70.08 ^b^	1.82 ^b^	2.04 ^b^	0.53 ^b^	1.93 ^a^	0.11
With Zn-Cr	1996.58 ^a^	72.30 ^a^	2.00 ^a^	2.33 ^a^	0.59 ^a^	1.61 ^b^	0.12
SEM	14.11	0.11	0.04	0.03	0.02	0.03	0.01
*p*-value	<0.0001	<0.0001	0.0078	<0.0001	0.0102	<0.0001	0.4426
	(B) Effect of organic Se levels						
Without Se	1820.66 ^b^	70.94 ^b^	1.92	2.11	0.55	1.82	0.11
With LSe ^≠^	1941.11 ^a^	71.42 ^a^	1.95	2.25	0.57	1.73	0.11
With HSe ^≠≠^	1956.21 ^a^	71.22 ^ab^	1.86	2.21	0.56	1.77	0.11
SEM	17.28	0.14	0.05	0.04	0.02	0.04	0.01
*p*-value	<0.0001	0.0745	0.4707	0.0646	0.7044	0.2610	0.9799
	(A × B) Effect of interaction between Zn-Cr mixture and organic Se levels						
T1	1704.28 ^d^	69.84 ^b^	1.80	1.89 ^c^	0.51	2.01 ^a^	0.10
T2	1937.05 ^b^	72.03 ^a^	2.04	2.32 ^a^	0.58	1.63 ^b^	0.12
T3	1840.45 ^c^	70.22 ^b^	1.86	2.11 ^b^	0.54	1.89 ^a^	0.11
T4	1901.53 ^cb^	70.18 ^b^	1.79	2.12 ^b^	0.53	1.91 ^a^	0.11
T5	2041.78 ^a^	72.62 ^a^	2.04	2.39 ^a^	0.60	1.57 ^b^	0.12
T6	2010.90 ^a^	72.27 ^a^	1.93	2.29 ^a^	0.59	1.63 ^b^	0.11
SEM	24.43	0.20	0.08	0.06	0.03	0.05	0.01
*p*-value	<0.0001	<0.0001	0.0992	<0.0001	0.1643	<0.0001	0.9632

^a,b,c,d^ = means with different letters in the same column are significantly different at (*p* < 0.05). SEM, standard error of the mean. ^≠^ LSe = low selenium concentration, ^≠≠^ HSe = high selenium concentration. T1 = (basal diet, no additive), T2 = T1 + 100 mg Zn + 1.5 mg Cr/Kg diet (Zn-Cr), T3 = T1 + 0.6 mg Se/Kg, T4 = T1 + 0.9 mg Se/Kg, T5 = T1 + (100 mg Zn + 1.5 mg Cr + 0.6 mg Se) and T6 = T1 + (100 mg Zn + 1.5 mgCr + 0.9 mg Se).

**Table 6:** Hematological parameters of broilers after dietary minerals (Zn-Cr and organic-Se) supplementation under heat stress conditions. (*n* = 8).

Treatments	Red Blood Cells, RBC (10^6^/mm^3^)	White Blood Cells, WBC (10^4^/mm^3^)	Haemoglobin, Hb (g/dL)	Packed Cell Volume, PCV (%)
(A) Effect of Zn-Cr mixture				
Without Zn-Cr	2.68 ^b^	3.38	8.55 ^b^	29.61 ^b^
With Zn-Cr	3.08 ^a^	3.51	9.29 ^a^	32.09 ^a^
SEM	0.05	0.05	0.14	0.34
*p*-value	<0.0001	0.1086	0.0018	<0.0001
(B) Effect of organic Se levels				
Without Se	2.83	3.40	8.59	30.76
With LSe ^≠^	2.87	3.39	9.12	30.69
With HSe ^≠≠^	2.94	3.53	9.06	31.10
SEM	0.07	0.07	0.18	0.41
*p*-value	0.5338	0.3240	0.0893	0.7473
(A × B) Effect of interaction between Zn-Cr mixture and organic Se levels				
T1	2.56 ^b^	3.27	8.11 ^b^	29.51 ^b^
T2	3.11 ^a^	3.54	9.06 ^a^	32.01 ^a^
T3	2.63 ^b^	3.37	8.82 ^ab^	29.28 ^b^
T4	2.85 ^ab^	3.49	8.73 ^ab^	30.03 ^b^
T5	3.10 ^a^	3.42	9.43 ^a^	32.09 ^a^
T6	3.03 ^a^	3.57	9.39 ^a^	32.18 ^a^
SEM	0.09	0.09	0.25	0.58
*p*-value	0.0011	0.2879	0.0145	0.0026

^a,b^ = means with different letters in the same column are significantly different at (*p* < 0.05). SEM, standard error of the mean. ^≠^ LSe = low selenium concentration, ^≠≠^ HSe = high selenium concentration. T1 = (basal diet, no additive), T2 = T1 + 100 mg Zn + 1.5 mg Cr/Kg diet (Zn-Cr), T3 = T1 + 0.6 mg Se/Kg, T4 = T1 + 0.9 mg Se/Kg, T5 = T1 + (100 mg Zn + 1.5 mg Cr + 0.6 mg Se) and T6 = T1 + (100 mg Zn + 1.5 mgCr + 0.9 mg Se).

**Table 7:** Serum blood biochemistry of broilers after dietary minerals (Zn-Cr and organic-Se) supplementation under heat stress conditions. (*n* = 8).

Treatments		g/dL	mg/dL						U/L		
	Total Protein	Albumin	Glucose	Triglycerides	Total Cholesterol	HDL-Cholesterol	LDL-Cholesterol	Creatinine	Uric Acid	ALT	AST
(A) Effect of Zn-Cr mixture											
Without Zn-Cr	3.67 ^b^	1.84 ^b^	185.77 ^a^	185.77 ^a^	149.32 ^a^	39.84 ^b^	99.39 ^a^	0.54 ^a^	7.89 ^a^	5.36	229.71
With Zn-Cr	4.03 ^a^	2.06 ^a^	153.33 ^b^	153.33 ^b^	135.86 ^b^	42.91 ^a^	81.05 ^b^	0.45 ^b^	6.26 ^b^	5.28	225.30
SEM	0.03	0.02	2.93	2.93	1.88	0.66	1.25	0.01	0.06	0.05	1.80
*p*-value	<0.0001	<0.0001	<0.0001	<0.0001	<0.0001	0.0040	<0.0001	0.0004	<0.0001	0.2712	0.1416
(B) Effect of organic Se levels											
Without Se	3.85	1.99	175.30	174.10 ^a^	144.76	40.30	94.73 ^a^	0.51	7.32 ^a^	228.27	5.33
With LSe ^≠^	3.86	1.94	168.39	163.51 ^b^	138.01	41.65	86.58 ^b^	0.49	6.97 ^b^	227.10	5.35
With HSe ^≠≠^	3.84	1.92	164.96	172.73 ^a^	145.00	42.18	89.35 ^b^	0.49	6.95 ^b^	227.90	5.28
SEM	0.04	0.03	3.59	2.96	2.31	0.81	1.53	0.02	0.08	2.21	0.06
*p*-value	0.9558	0.2571	0.1453	0.0424	0.0771	0.2607	0.0047	0.7412	0.0045	0.7213	0.9291
(A × B) Effect of interaction between Zn-Cr mixture and organic Se levels											
T1	3.70 ^b^	1.85 ^cb^	195.56 ^a^	190.29 ^a^	156.63 ^a^	39.56 ^b^	109.45 ^a^	0.55 ^a^	8.08 ^a^	230.42	5.40
T2	4.01 ^a^	2.12 ^a^	155.05 ^c^	157.92 ^b^	132.89 ^c^	41.03 ^b^	80.01 ^d^	0.46 ^bc^	6.55 ^c^	226.12	5.26
T3	3.62 ^b^	1.79 ^c^	186.79 ^ab^	178.70 ^a^	145.81 ^b^	40.41 ^b^	96.83 ^b^	0.52 ^ab^	7.95 ^ab^	230.03	5.41
T4	3.69 ^b^	1.89 ^cb^	174.96 ^b^	185.76 ^a^	145.50 ^b^	39.54 ^b^	91.88 ^cb^	0.55 ^a^	7.64 ^b^	228.68	5.28
T5	4.09 ^a^	2.09 ^a^	150.00 ^c^	148.32 ^b^	130.21 ^c^	42.88 ^ab^	76.32 ^d^	0.46 ^bc^	5.99 ^d^	224.16	5.29
T6	4.00 ^a^	1.96 ^b^	154.95 ^c^	159.70 ^b^	144.50 ^b^	44.82 ^a^	86.82 ^c^	0.42 ^c^	6.25 ^cd^	227.11	5.29
SEM	0.06	0.04	5.08	4.19	3.26	1.14	2.16	0.02	0.11	3.12	0.09
*p*-value	<0.0001	<0.0001	<0.0001	<0.0001	0.0002	0.0258	<0.0001	0.0092	<0.0001	0.7385	0.7009

^a,b,c,d^ = means with different letters in the same column are significantly different at (*p* < 0.05). SEM, standard error of the mean. ^≠^ LSe = low selenium concentration, ^≠≠^ HSe = high selenium concentration. T1 = (basal diet, no additive), T2 = T1 + 100 mg Zn + 1.5 mg Cr/Kg diet (Zn-Cr), T3 = T1 + 0.6 mg Se/Kg, T4 = T1 + 0.9 mg Se/Kg, T5 = T1 + (100 mg Zn + 1.5 mg Cr + 0.6 mg Se) and T6 = T1 + (100 mg Zn + 1.5 mgCr + 0.9 mg Se).

**Table 8:** Thyroid hormones of broilers after dietary minerals (Zn-Cr and Se) supplementation under heat stress conditions. (*n* = 8).

Treatments	Triiodothyronine (T3), ng/mL)	Thyroxine (T4), ng/mL
(A) Effect of Zn-Cr mixture		
Without Zn-Cr	2.01 ^b^	8.45
With Zn-Cr	2.20 ^a^	8.88
SEM	0.03	0.15
*p*-value	0.0012	0.0544
(B) Effect of organic Se levels		
Without Se	2.02 ^b^	8.35
With LSe ^≠^	2.18 ^a^	8.77
With HSe ^≠≠^	2.11 ^ab^	8.88
SEM	0.07	0.18
*p*-value	0.0390	0.1160
(A × B) Effect of interaction between Zn-Cr mixture and organic Se levels		
T1	1.97 ^b^	8.29
T2	2.07 ^b^	8.41
T3	1.98 ^b^	8.55
T4	2.09 ^b^	8.52
T5	2.39 ^a^	9.00
T6	2.14 ^b^	9.24
SEM	0.06	0.25
*p*-value	0.0011	0.1122

^a,b^ = means with different letters in the same column are significantly different at (*p* < 0.05). SEM, standard error of the mean. ^≠^ LSe = low selenium concentration, ^≠≠^ HSe = high selenium concentration. T1 = (basal diet, no additive), T2 = T1 + 100 mg Zn + 1.5 mg Cr/Kg diet (Zn-Cr), T3 = T1 + 0.6 mg Se/Kg, T4 = T1 + 0.9 mg Se/Kg, T5 = T1 + (100 mg Zn + 1.5 mg Cr + 0.6 mg Se) and T6 = T1 + (100 mg Zn + 1.5 mgCr + 0.9 mg Se).

**Table 9:** Serum antioxidant activity of broilers after dietary minerals (Zn-Cr and Se) supplementation under heat stress conditions. (*n* = 8).

Treatments	Total Antioxidant Capacity (TAC), mM/L	Malondialdehyde (MDA), nmol/mL
(A) Effect of Zn-Cr mixture		
Without Zn-Cr	0.61 ^b^	1.87
With Zn-Cr	0.71 ^a^	1.72
SEM	0.03	0.06
*p*-value	0.0088	0.0657
(B) Effect of organic Se levels		
Without Se	0.59 ^b^	1.99 ^a^
With LSe ^≠^	0.66 ^ab^	1.67 ^b^
With HSe ^≠≠^	0.73 ^a^	1.72 ^b^
SEM	0.03	0.007
*p*-value	0.0145	0.0083
(A × B) Effect of interaction between Zn-Cr mixture and organic Se levels		
T1	0.50 ^b^	3.23 ^a^
T2	0.69 ^a^	2.75 ^b^
T3	0.62 ^ab^	2.70 ^b^
T4	0.72 ^a^	2.68 ^b^
T5	0.71 ^a^	2.65 ^b^
T6	0.75 ^a^	2.76 ^b^
SEM	0.04	0.10
*p*-value	0.0077	0.0041

^a,b^ = means with different letters in the same column are significantly different at (*p* < 0.05). SEM, standard error of the mean. ^≠^ LSe = low selenium concentration, ^≠≠^ HSe = high selenium concentration. T1 = (basal diet, no additive), T2 = T1 + 100 mg Zn + 1.5 mg Cr/Kg diet (Zn-Cr), T3 = T1 + 0.6 mg Se/Kg, T4 = T1 + 0.9 mg Se/Kg, T5 = T1 + (100 mg Zn + 1.5 mg Cr + 0.6 mg Se) and T6 = T1 + (100 mg Zn + 1.5 mgCr + 0.9 mg Se).

## Data Availability

The data that support the findings of this study are available on request from the corresponding author.
